# Bioactive Coatings Loaded with Osteogenic Protein for Metallic Implants

**DOI:** 10.3390/polym13244303

**Published:** 2021-12-09

**Authors:** Oana Gherasim, Alexandru Mihai Grumezescu, Valentina Grumezescu, Ecaterina Andronescu, Irina Negut, Alexandra Cătălina Bîrcă, Bianca Gălățeanu, Ariana Hudiță

**Affiliations:** 1 Department of Science and Engineering of Oxide Materials and Nanomaterials, Faculty of Applied Chemistry and Materials Science, Politehnica University of Bucharest, 011061 Bucharest, Romania; oana.fufa@gmail.com (O.G.); grumezescu@yahoo.com (A.M.G.); ecaterina.andronescu@upb.ro (E.A.); ada_birca@yahoo.com (A.C.B.); 2 Lasers Department, National Institute for Lasers, Plasma, and Radiation Physics, RO-77125 Magurele, Romania; negut.irina@inflpr.ro; 3 Academy of Romanian Scientists, Ilfov No. 3, 50044 Bucharest, Romania; 4 Research Institute of the University of Bucharest—ICUB, University of Bucharest, 050657 Bucharest, Romania; 5 Department of Biochemistry and Molecular Biology, Faculty of Biology, University of Bucharest, 91–95 Splaiul Independentei, 050095 Bucharest, Romania; bianca.galateanu@bio.unibuc.ro (B.G.); arianahudita@yahoo.com (A.H.)

**Keywords:** hydroxyapatite, bone morphogenetic protein, MAPLE

## Abstract

Osteoconductive and osteoinductive coatings represent attractive and tunable strategies towards the enhanced biomechanics and osseointegration of metallic implants, providing accurate local modulation of bone-to-implant interface. Composite materials based on polylactide (PLA) and hydroxyapatite (HAp) are proved beneficial substrates for the modulation of bone cells’ development, being suitable mechanical supports for the repair and regeneration of bone tissue. Moreover, the addition of osteogenic proteins represents the next step towards the fabrication of advanced biomaterials for hard tissue engineering applications, as their regulatory mechanisms beneficially contribute to the new bone formation. In this respect, laser-processed composites, based on PLA, Hap, and bone morphogenetic protein 4(BMP4), are herein proposed as bioactive coatings for metallic implants. The nanostructured coatings proved superior ability to promote the adhesion, viability, and proliferation of osteoprogenitor cells, without affecting their normal development and further sustaining the osteogenic differentiation of the cells. Our results are complementary to previous studies regarding the successful use of chemically BMP-modified biomaterials in orthopedic and orthodontic applications.

## 1. Introduction

Besides biomechanical support and functional performance, surface characteristics of metallic implants are essential aspects to be considered during the fabrication of implantable devices for orthopedic and orthodontic applications. It has been shown that specific surface features of metallic implants are responsible for their long-term mechanical stability and tribological outcomes [1,2,3], corrosion resistance, and potential ion-mediated toxicity [4,5,6], as well as for their osseointegration (by influencing the behavior of cells that control the final bone formation) [7,8,9]. As metallic biomaterials represent key elements for hard tissue replacement and restoration, a plethora of efforts have been undertaken to limit their main shortcomings, namely bioinertness and poor bioactivity. Many studies confirmed the importance of surface characteristics for reaching a proper and augmented osseointegration [10,11,12,13], with a special emphasis forming a bioactive implant-to-bone interface.

Surface modification of metallic implants, either performed by mechanical, chemical, or physical techniques [14,15,16], results in beneficial outcomes in implants’ reactivity, hydrophilicity, roughness, surface energy and charge, and, especially, biocompatibility. The physical methods that have been applied to enhance the osteogenic activity of implantable metallic biomaterials include thermal spraying [17], sputtering [18], ion implantation [19], plasma treatment [20], and laser-assisted processing [21]. Special attention was oriented towards the latter category, given the enhanced physicochemical properties and boosted biofunctional performance of laser-textured [22,23,24] and coated [25,26] metallic implants. Out of all these techniques, the matrix-assisted pulsed laser evaporation (MAPLE) is known to have some advantages, such as the possibility to synthesize homogeneous and uniform nanosized or nanostructured coatings, as well as composite or hybrid coatings containing organic substances, such as polymers [27,28,29] and biomolecules [30,31].

Surface modification by calcium phosphate coatings, obtained by laser processing, represents a suitable choice to increase the integration of metallic implants and improve their biofunctionality [32,33]. Synthetic hydroxyapatite (HAp) is one of the most widely utilized calcium phosphate biomaterials for bone tissue engineering applications, as it shows chemical and structural resemblance with the biological apatite [34,35] and excellent biocompatibility [34]. Synthetic HAp is responsible for an increased concentration of local Ca^2+^, which can further stimulate osteoblasts proliferation and encourage the growth and differentiation of mesenchymal stem cells [36]. HAp-based laser-processed coatings demonstrated high efficiency for osseointegration of metallic implants and subsequent bone regeneration [21,34].

In addition, the HAp-aided osseointegration of metallic materials can be further enhanced by developing composite or hybrid coatings, as the presence of different ions [37,38,39] and nanostructures [40,41] has beneficial effects on the crystallization, mechanical properties, degradation, and biological activity of apatite and enhances the repair mechanisms of bones.

Polylactic acid (PLA) is a linear aliphatic polyester with good mechanical and thermoplastic properties, superior solubility and degradation, excellent biocompatibility, and tunable biodegradability [42,43,44]. It has been widely explored for biomedical applications, including surgical sutures, implants, bone grafts, and drug carriers [45,46,47]. In addition, PLA coatings were reported as successful platforms for the circumstantial release of bioactive molecules [48,49]. Given the physicochemical versatility and biological behavior of PLA/HAp composites, as well as their ability to improve the osteogenic response of metallic biomaterials both in vitro [50] and in vivo [51], the synthesis of PLA/HAp composite coatings for enhanced osseointegration of metallic implants the focus of several papers [52,53].

Moreover, the immobilization or incorporation of growth factors, with an essential role during the repair and regeneration of bone tissue, is an attractive strategy to promote and support the osseointegration of metallic implants and subsequent bone healing. Several growth factors have been explored for their capacity to advance the healing process of bone tissue, but bone morphogenetic proteins (BMPs) are among the most proficient. Those multifunctional cytokines play a pivotal role in bone remodeling, both during osteoblastogenesis and osteoclast homeostasis [54,55]. BMPs are extensively explored for orthopedics and oral maxillofacial surgery [56,57,58]. Advanced biomaterials based on osteoinductive calcium phosphates, such as HAp [59,60] and tricalcium phosphate [61], were reported as effective delivery platforms for osteogenic BMPs.

Immobilizing or embedding osteoinductive proteins from the BMP family on/in implantable materials and devices used in orthopedics and orthodontics is an effective approach to increasing biomechanical and functional performance. In this regard, the superior efficacy of supporting cell proliferation and inducing osteogenic differentiation of stem cells in fibroin/HAp scaffolds [62], nano-HAp/collagen/PLA [63], and PCL/HAp [64], enriched with BMP2, have been reported. The osteogenic potential, as well as the capacity to simulate *de novo* bone tissue formation, has also been reported when incorporating plasmid-activated BMP2-loaded chitosan nanoparticles into collagen/HAp scaffolds [65]. More, the potential of BMP2-loaded nano-HAp/PLA-PEG composites [66] and hybrid PLA/HAp nanofibrillary structures, decorated with BMP2-loaded liposomal nanocapsules [66], to be used in bone engineering, has also been highlighted. The potential of BMP2-loaded nano-HAp/PLA-PEG composites and hybrid PLA/HAp nanofibrillary structures, decorated with BMP2-loaded liposomal nanocapsules [67], to be used in bone engineering, has been supported by the multifunctionality of these materials, which possess the ability of biomineralization and stimulate osteogenic differentiation and generation of new bone tissue. Prolonged and controlled release of BMP2 from three-dimensional biomimetic supports, based on HAp/collagen [68,69] and HAp/chitosan [70,71], has accelerated the process of bone regeneration; these advanced materials also demonstrating the ability to stimulate angiogenesis and neovascularization.

With the aim to improve the bioactivity of metallic implants, we herein evaluated the ability of MAPLE-obtained composite coatings, based on PLA, Hap, and BMP4 (PLA/HAp/BMP4), to modulate the complex response of osteoprogenitor cells. In this view, the PLA/HAp/BMP4 materials samples were synthetized and characterized, in terms of their physicochemical properties and morphology by XRD, SEM, FT-IR, and IRM. More, the samples were subjected to a set of biological tests, revealing their level of biocompatibility and potential to sustain pre-osteoblasts differentiation towards the osteogenic lineage.

## 2. Materials and Methods

### 2.1. Materials

All reagents required for the synthesis of HAp-based composite coatings were purchased from Sigma-Aldrich (Merck Group, Darmstadt, Germany), namely polylactic acid (PLA), CaCl_2,_ Na_2_HPO_4_·2H_2_O, NaOH (10%), dimethyl sulfoxide (DMSO), and bone morphogenetic protein 4 (BMP4).

The same supplier provided most reagents and assay kits used for biological evaluation (otherwise, the provider was specified below). The MC3T3-E1 osteoblastic cell line, derived from mouse calvaria (ATCC^®^ CRL-2593™), was acquired from American Type Culture Collection (ATCC, Manassas, VA, USA).

### 2.2. HAp Synthesis

To synthesize the HAp powdery sample, CaCl_2_ and Na_2_HPO_4_ × 2H_2_O were dissolved in ultrapure water. The phosphorous-containing solution was then added dropwise to the calcium-containing solution, under continuous stirring. Subsequently, the alkaline pH adjustment was performed by adding 10% NaOH, and the resulted solution underwent a one-day maturation process. The final product was subjected to filtration, triple washing treatment, and drying process.

### 2.3. Composite Coatings Synthesis

Titanium discs (with diameter and thickness of 12 mm and 0.1 mm, respectively), and double-side polished (1 0 0) Si slides were used as substrates during the MAPLE experiments. Prior to surface modification by laser processing, all substrates were subjected to an ultrasonic cleaning treatment with acetone, ethanol, and deionized water (15 min each step), followed by drying under a high purity nitrogen jet.

The solid targets required for MAPLE experiments were obtained by freezing the PLA/HAp/BMP4 suspensions in DMSO (3% concentration) at liquid nitrogen temperature. Further, the targets were irradiated with a COMPexPro 205 Lambda Physics KrF* excimer laser source (λ = 248 nm, τFWHM = 25 ns), at a repetition rate of 15 Hz and a residual pressure of 3 × 10^−3^ mbar. The target-to-substrate distance was set at 5 cm. A total number of ~58,000 laser pulses were applied for each experiment, for depositions using different laser fluences (200, 300, and 400 mJ/cm^2^).

### 2.4. Characterization Methods

The X-ray diffraction (XRD) analyses were performed using the Cu_Kα_ radiation (λ = 1.056 Å) of an XRD-6000 Shimadzu equipment (Duisburg, Germany). Data were collected between 10–80° diffraction angles.

SEM investigations were performed on HAp powder and HAp-based coatings, using the secondary electron beams (30 keV and 25 keV, respectively) of an Inspect S scanning electronic microscope from FEI (Thermo Fischer Scientific, Hillsboro, OR, USA). Before SEM analysis, all samples were capped with a thin conductive layer.

Fourier transform infrared spectroscopy (FT-IR) spectra of HAp powder and composite coatings, as well as infrared microscopy (IRM) maps of Hap-based coatings, were recorded on a Nicolet iN10 MX FT-IR microscope (Thermo Fischer Scientific, Waltham, MA, USA), equipped with an MCT liquid nitrogen cooled detector. The measurements, performed in the reflection mode, were collected in the 4000–600 cm^−1^ wavenumber range, at 4 cm^−1^ resolution. Multiple scans were co-added and converted to absorbance, in order to attain each IR spectrum, using in this respect the Ominc Picta software (Thermo Fischer Scientific Company, Waltham, MA, USA).

### 2.5. Biological Evaluation

#### 2.5.1. Cell Culture Model

Mouse pre-osteoblasts from the MC3T3-E1 cell line (CRL-2593, ATCC) were used as in vitro cell culture model to evaluate the biocompatibility of PLA/HAp/BMP4 materials, as well as for assessing the osteogenic differentiation potential of the proposed material. Before cell seeding, all the tested composites were sterilized by exposure to UV light. Besides the PLA/HAp/BMP4 materials, non-coated substrates (Titan discs) were employed as reference material and were processed identically as the PLA/HAp/BMP4-coated samples.

For the biocompatibility assessment, the MC3T3-E1 preosteoblasts cells were seeded at an initial density of 1 × 10^4^ cells/cm^2^ on the surface of the samples in Dulbecco’s modified Eagle medium (DMEM, Sigma-Aldrich), supplemented with 10% fetal bovine serum (FBS, Life Technologies, Foster City, CA, USA) and 1% penicillin/streptomycin mixture (10,000 units/mL penicillin and 10 mg/mL streptomycin) (Sigma-Aldrich) and maintained under standard culture conditions (37 °C, 5% CO_2_) for 7 days. During the 7 days of maintaining the bioconstructs in culture, the cell culture media was refreshed every other day. For the osteogenic differentiation assessment, the MC3T3-E1 pre-osteoblasts cells were seeded at an initial density of 2 × 10^4^ cells/cm^2^ on the surface of the samples in complete DMEM culture media. After 24 h, the cell culture media was replaced with a commercially available osteogenic induction culture medium (StemPro Osteogenic Differentiation Kit, Thermo Fischer Scientific), and the samples were maintained in culture for 21 days under standard culture conditions. The osteogenic induction culture medium was refreshed three times a week.

#### 2.5.2. In Vitro Biocompatibility Assessment

The PLA/HAp/BMP4 materials biocompatibility was investigated 2 and 7 days after the achievement of the cell/composite bioconstructs, by evaluating the MC3T3-E1 cell viability and proliferation potential as the material cytotoxicity and impact on cell morphology.

The potential of the PLA/HAp/BMP4 materials to sustain cell viability and proliferation, as well as the pattern of cell distribution on the material surface, was investigated by the quantitative live/dead fluorescence microscopy assay, using the Live/Dead kit (Life Technologies). Briefly, the samples were retrieved from the cell culture media, rinsed with phosphate saline buffer (PBS, Life Technologies, Foster City, CA, USA), and incubated in the freshly prepared staining solution, containing both calceinAM and ethidium bromide, according to the manufacturer’s recommendations. After 20 min of incubation, at room temperature in darkness, the samples were investigated using the Olympus IX73 (Olympus Life Science, Waltham, MA, USA) microscope, equipped with a fluorescence modulus.

To quantitatively investigate the cell viability and proliferation of MC3T3-E1, seeded in contact with the PLA/HAp/BMP4 materials, the cell metabolic activity was evaluated using the MTT assay. For this, the cell culture media was discarded and replaced with 1 mg/mL 3-(4,5-dimethyldiazol-2-yl)-2,5-diphenyltetrazolium bromide MTT solution (Sigma-Aldrich), freshly prepared in FBS-free culture media. After 4 h of incubation at 37 °C, the MTT solution was removed, and the resulting formazan crystals were dissolved in DMSO. The absorbance of the resulting solution was measured at 550 nm using a FlexStation III microplate multimodal reader (Molecular Devices, San Jose, CA, USA).

Evaluation of the cytotoxicity of the PLA/HAp/BMP4 materials was performed via LDH assay using the lactate dehydrogenase (LDH), based in vitro toxicology assay kit (TOX7 kit, Sigma-Aldrich). Cell culture media samples were harvested at both experimental time points and mixed with the kit’s components, according to the manufacturer’s indications. The resulting solutions were incubated at room temperature, for 20 min in the dark, and the reaction was stopped with 1 N HCl. The LDH enzyme’s activity in the culture media was determined by measuring at 490 nm the absorbance of the resulting solutions, using the FlexStation III microplate multimodal reader (Molecular Devices).

To reveal the morphology of MC3T3-E1, cultured in contact with the PLA/HAp/BMP4 materials, the adhered cells were fixed with a 4% paraformaldehyde solution (PFA, Sigma-Aldrich) for 20 min, permeabilized with a 2% BSA solution with 0.1% Triton X100 (Sigma-Aldrich) for 1 h, and further stained with fluorescein isothiocyanate (FITC)-conjugated phalloidin (Sigma-Aldrich) for 1 h at 37 °C in the dark. Before samples microscopy investigation, using the Olympus IX73 fluorescent microscope, the cell nuclei were stained with 4, 6-diamidino-2-phenylindole (DAPI, Sigma-Aldrich).

#### 2.5.3. In Vitro Osteoinductive Potential Assessment

The osteogenic differentiation process was monitored, during 21 days of exposure of MC3T3-E1 cells, cultured on the surface of PLA/HAp/BMP4 materials, in the presence of osteogenic inductors, at 2-time points: 14 and 21 days.

The protein expression levels of the osteogenic-specific markers osteopontin (OPN) and osteocalcin (OCN) were investigated by fluorescence microscopy, following specific antibody staining. In this view, samples were fixed and permeabilized, as described above, and incubated overnight at 4 °C, with rabbit polyclonal anti- OCN (Santa-Cruz Biotechnology, Heidelberg, Germany) and goat polyclonal anti-OPN (Santa-Cruz Biotechnology, Heidelberg, Germany) antibodies. Prior to fluorescence microscopy investigation, the samples were further incubated in tetramethylrodamine-5,6-isothiocyanate (TRITC)-conjugated goat anti-rabbit and FITC-conjugated rabbit anti-goat secondary antibodies solutions for 30 min at room temperature in darkness (Santa-Cruz Biotechnology, Heidelberg, Germany) and DAPI for nuclei staining.

Alkaline phosphatase (ALP) activity was evaluated using the Alkaline Phosphatase Activity Colorimetric Assay Kit (Biovision, Milpitas, CA, USA), following the manufacturer’s instructions. At the chosen time points, for monitoring the osteogenic induction, the culture media was harvested, mixed with p-nitrophenylphosphate substrate, and incubated at 25 °C for 60 min. The optical density of the resultant p-nitrophenol at 405 nm was determined spectrophotometrically, and the results were plotted on the p-nitrophenol standard curve to quantify the amount of pNP generated by ALP in each sample. ALP activity was determined as described in the kit protocol.

To evaluate the capacity of MC3T3-E1 cells to form calcium deposits in contact with the PLA/HAp/BMP4 materials, the alizarin red S staining was employed. Briefly, at the experimental time points, the cell culture media was discarded, and samples were further washed with PBS and fixed with 4% PFA for 2 h. For staining, a freshly prepared solution of 1% Alizarin Red (Sigma-Aldrich) was used for immersing the samples for 30 min at room temperature. Then, the dye was removed, and the samples were washed with distilled water, until the washing solution remained colorless and transferred in a 10% acetic acid solution for dye extraction. The optical density of the resulting solutions was determined spectrophotometrically at 405 nm using a Flex Station III multimodal reader (Molecular Devices).

#### 2.5.4. Statistical Analysis

Statistical analyses were carried out using GraphPad Prism Software (San Diego, CA, USA). All statistic data are presented as mean values ± standard deviation of three independent experiments. Both one- and two-way analyses of variance (ANOVA) were used. Bonferroni’s multiple comparisons post-test were used to identify which groups were different, with *p* < 0.05 considered statistically significant. For the microscopy-based assays, image acquisition and processing were performed using dedicated software (CellSense F, Olympus and Image J, National Institutes of Health, Bethesda, MD, USA).

## 3. Results & Discussion

### 3.1. Physicochemical Characterization of HAp Powder

**Figure 1:**
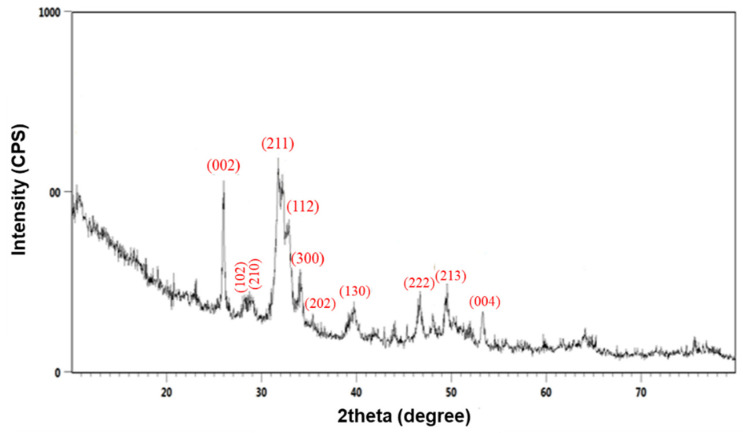
XRD pattern of HAp powder.

Following the complete drying of the viscous white precipitate, resulted from chemical synthesis, XRD analysis was performed, the corresponding results being included in Figure 1.

Diffraction maxima corresponding to the hexagonal crystalline structure of hydroxyapatite were identified at 2θ values of 26.4°, 28.7°, 29.4°, 32.2°, 33.9°, 34.1, 35.6°, 39.9°, 47.6°, 49.9°, and 53.6°. The corresponding diffraction planes are evidenced in Figure 1. The intense doublet, attributed to the presence of (211) and (112) planes, confirmed the synthesis of stoichiometric crystalline HAp [72], according to the JCPDS09-0432 file and previous results from the literature [73,74].

The presence of large, overlapped reflection peaks indicated the reduced crystallinity of the synthesized material, a result expected due to the absence of additional thermal treatments [75,76]. However, the formation of high purity HAp powder was confirmed, as no secondary phases were noticed.

**Figure 2:**
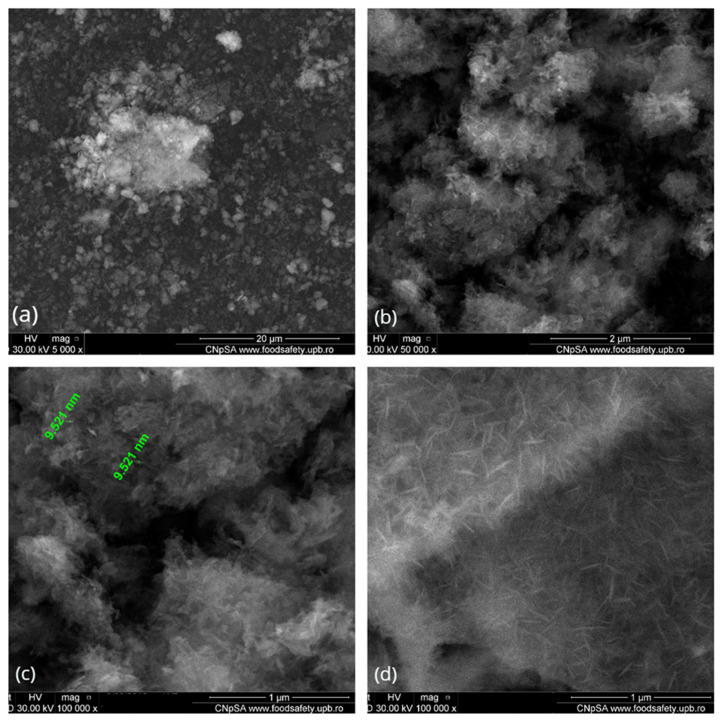
SEM images of HAp powder at (**a**) 5000×, (**b**) 10,000× and (**c**,**d**)100,000 × magnifications.

The SEM measurements (Figure 2) showed the presence of HAp aggregates, consisting in needle-like nanosized individual particles (width of ~10 nm, length between 10−100 nm). Those observations are in good agreement with other reports on the microstructure of synthetic HAp [77,78].

**Figure 3:**
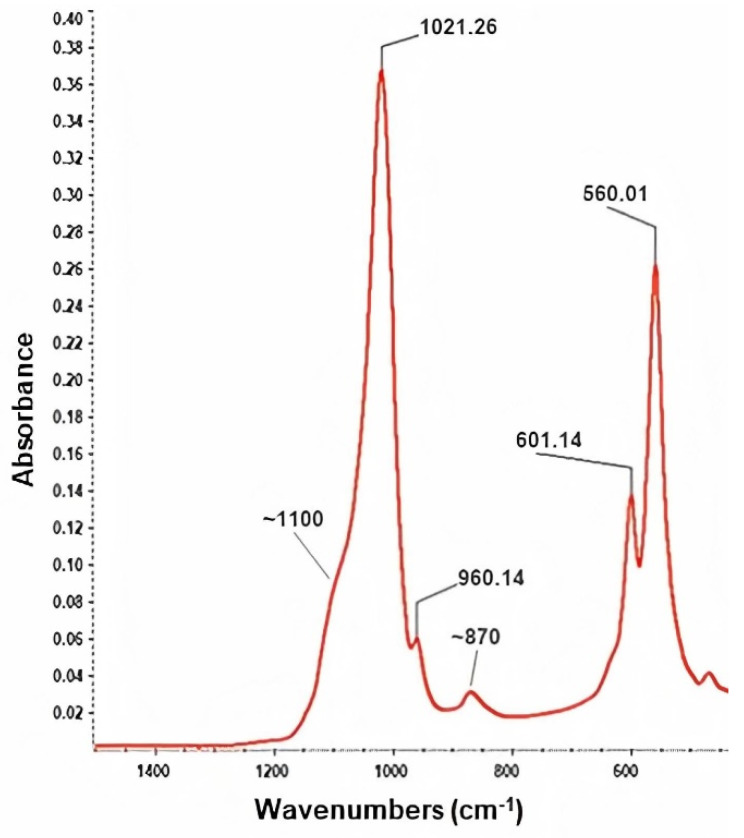
IR spectrum of HAp powder.

The IR spectrum of the HAp powder (Figure 3) revealed the presence of the following characteristic absorption bands: the intense peaks from 1021 cm^−1^ and 1100 cm^−1^, attributed to the asymmetric stretching of PO_4_^3−^ group (P–O bond), while the symmetric stretch of the same functional group was identified at 960 cm^−1^ and 870 cm^–1^ [79,80]. Moreover, the asymmetric deformation of the O−P−O bond (originating from PO_4_^3−^) was confirmed by the presence of IR bands from ~601 cm^−1^ and ~560 cm^−1^ [81,82]. The particular presence of the latter doublet is characteristic for crystalline HAp [83], in compliance with the XRD results.

### 3.2. Physicochemical Characterization of PLA/HAp/BMP4 Coatings

**Figure 4:**
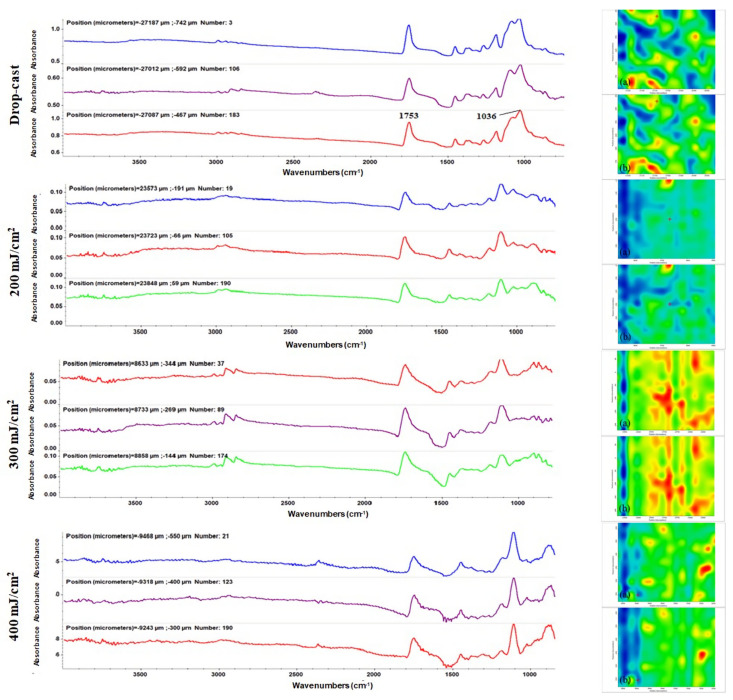
IR spectra (**left**) and IR maps (**right**), corresponding to the distribution of PLA-originating methylene (**a_1_**–**a_4_**) and HAp-originating phosphate (**b_1_**–**b_4_**), collected for PLA/HAp/BMP4 coatings.

To experimentally identify the optimal conditions for the unaltered and efficient transfer of MAPLE-processed coatings, compositional and microstructural studies are often required. In literature, there are reported a wide array of techniques for the fabrication of BMP related films [84,85]; however, the control and repeatability of the process are known as one of the main bottlenecks of solution-based approaches. For the transfer of complex organic material with controllable stoichiometries laser-based technologies were shown to be desirable alternatives. In our case, comparative IR investigations between drop-cast (corresponding to pristine materials) and MAPLE-processed samples (obtained with different laser fluences) were performed (Figure 4). In this respect, IR maps (Figure 4-righthand side) were recorded by monitoring the intensity of the absorption bands of the carbonyl group (~1750 cm^−1^), which originates from the polyester and phosphate groups (~1030 cm^−1^) from the apatite phase. The chromatic changes, seen in the IR maps, are directly related to absorbance intensity and provide valuable information on the compositional distribution across the substrate and the efficiency of the laser transfer. Complementary IR spectra (Figure 4-lefthand side), resulting from collecting data from different points on the samples, offered information on the compositional integrity and stoichiometry of the MAPLE-processed materials.

Besides the characteristic absorbance maxima of HAp (identified at ~1100, ~1036 and ~980 cm^−1^), IR maxima corresponding to PLA were clearly seen and assigned as follows: symmetric and asymmetric stretching of C–H band originating from –CH_3_ terminal groups (between 3000–2850 cm^−1^), strong stretching of C=O moiety(~1750 cm^−1^), asymmetric deformation of C–H (~1450 cm^−1^), –CH_3_ bending (~1380 cm^−1^), C–O–C asymmetric stretching (~1200 cm^−1^), and C–O stretch vibrations (~1100 cm^−1^) [86,87]. To further identify the optimal laser fluence for processing the PLA/HAp/BMP4 materials, the IR spectra of drop-cast samples were used as reference. A significant reduction (even disappearance) of relevant absorption bands was observed for samples processed with minimal and maximal laser fluence values. This observation was correlated with the poor transfer of composite material that occurred at low fluence (200 mJ/cm^2^) or with the nonstoichiometric transfer of composite material that occurred at high fluence (400 mJ/cm^2^), respectively. Those results were supported by the IR mapping, which revealed the abundance of cool colored areas. In terms of preserved stoichiometry and laser transfer efficiency, optimal results were found for materials processed with the average laser fluence of 300 mJ/cm^2^.

**Figure 5:**
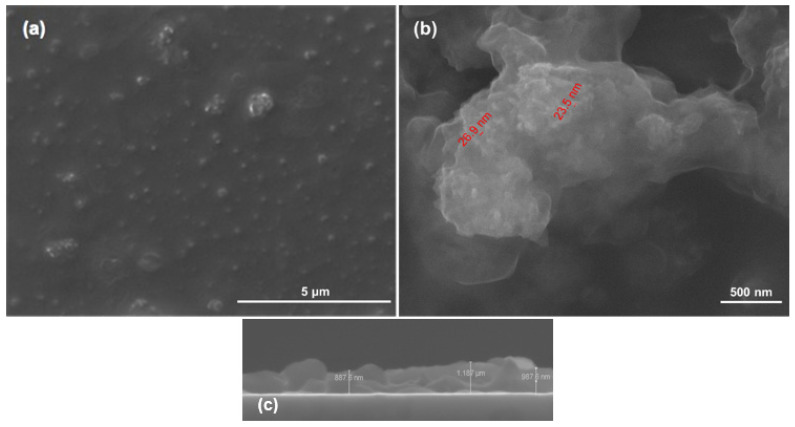
Top-view (**a**,**b**) and cross-section (**c**) SEM images of PLA/HAp/BMP4 coatings, obtained at 300 mJ/cm^2^ laser fluence.

Based on the results provided by IR analysis, the following investigations were performed only on PLA/Hap/BMP4 materials processed at 300 mJ/cm^2^ laser fluence. Relevant microstructural information was obtained by both top-view and cross-section SEM micrographs (Figure 5). Figure 5a revealed the uniform and compact transfer of composite material onto the substrate, with HAp aggregates uniformly distributed in the polymer matrix. At this level, no degradation of PLA film was noticed, confirming the conclusions extracted from IR measurements. Figure 5b evidenced the efficient incorporation of HAp nanoparticles within the PLA matrix, as they preserved their dimensional range during the MAPLE transfer. Moreover, the preferential rod shape of HAp was observed at this level, which was correlated with the individual coating of inorganic nanoparticles by the polymer matrix. This observation was related to the formation of weak physical interactions between the two components, as previously evidenced by the spectral shift of HAp absorption bands in the MAPLE-processed materials. As observed from SEM investigations, the laser fluence did not alter the uniform distribution of small aggregates of PLA/HAp/BMP4 composite material onto the substrate. The continuous sub-micron coating (Figure 5c) was composed of particulates of different sizes, arbitrary scattered on the surface, which have a positive outcome on cell adhesion and growth/proliferation [88,89,90].

### 3.3. In Vitro Biocompatibility of PLA/HAp/BMP4 Coatings

The biocompatibility of the PLA/HAp/BMP4 coatings was investigated by multiple assays that aimed to highlight the affinity of the MC3T3-E1 preosteoblasts cells to adhere to the nanostructured surfaces, as well as the capacity of the materials to sustain cell viability and proliferation.

**Figure 6:**
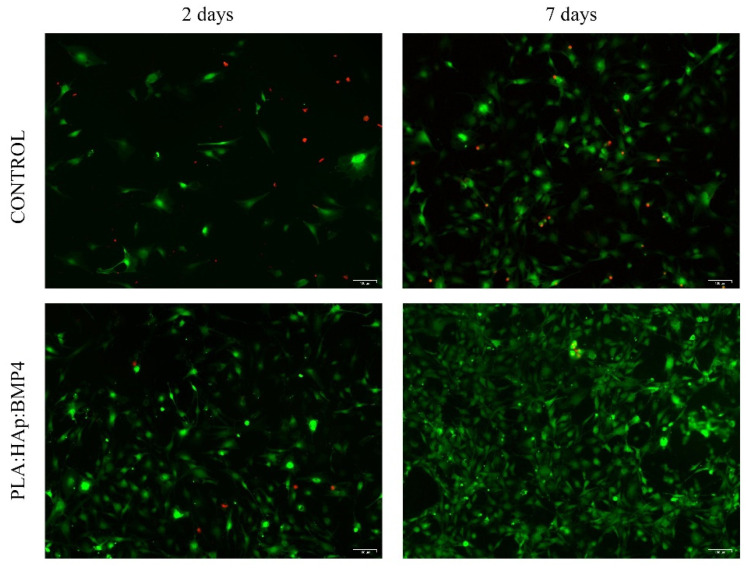
Fluorescence micrographs of live (green) and dead (red) MC3T3-E1 cells, after 2 and 7 days of culture on the surface of the control and PLA/HAp/BMP4 materials (scale bar 100 µm).

Fluorescence microscopy investigation of the samples after live/dead staining (Figure 6) showed that PLA/HAp/BMP4 coatings increase the affinity of MC3T3-E1 cells significantly to adhere to the substrate, as revealed by the enhanced ratio of cells present on the PLA/Hap/BMP4 material, 2 days post-seeding, as compared with the control. The cells were well-distributed on the entire surface of the material, being identified exclusively cells labeled in green (live cells). This particularity was maintained as well after 7 days of culture, where MC3T3-E1 cells uniformly covered the entire surface of the PLA/Hap/BMP4 material, unlike the control substrate, where a significantly lower ratio of viable cells was identified onto the control surface. Even if both substrates provide support for MC3T3-E1 preosteoblasts cell proliferation, the reduced number of cells present on the reference sample, as compared with the PLA/Hap/BMP4 coating, shows that the substrate tuning is mandatory to promote cellular adhesion and maintain overtime cellular health.

**Figure 7:**
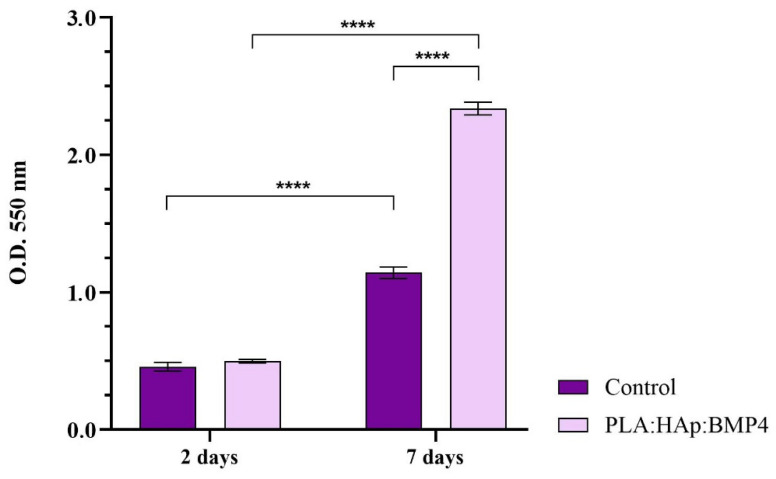
MC3T3-E1 cell viability and proliferation potential, after 2 and 7 days of culture of contact with control surfaces and PLA/HAp/BMP4 materials, as revealed by the MTT assay (**** *p* < 0.0001).

The observations made via fluorescence microscopy were confirmed by the MTT spectrophotometric assay that allowed the quantification of the viable cells cultured on the surface of the sample, as well as the preosteoblasts proliferation rate (Figure 7). After 2 days of culture, no significant changes were observed between samples, in terms of cell viability. In contrast, after 7 days of cell-materials interaction, the metabolic activity of MC3T3-E1 cells was significantly increased in contact with the nanostructured substrates, compared with the pristine substrates. More, while the cell viability of MC3T3-E1 cells, after 2 days of culture, was similar on both samples, after 7 days, a 2-fold increase of the cell viability was observed between samples, confirming that the PLA/Hap/BMP4 coating augments MC3T3-E1 cell proliferation.

**Figure 8:**
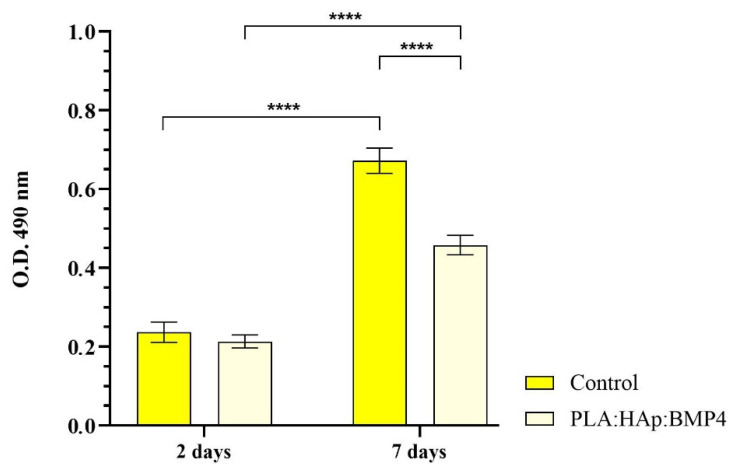
Quantification of LDH leakage, triggered by MC3T3-E1 cell membrane damage, as a measure of samples cytotoxicity after 2 and 7 days of culture (**** *p* < 0.0001) of contact with the control surface and PLA/HAp/BMP4 coating.

More, LDH release in the culture media was quantified spectrophotometrically to assess the cytotoxic potential of the investigated samples (Figure 8). After 2 days of culture, MC3T3-E1 pre-osteoblasts grown on the sample’s surfaces displayed a low LDH release in the culture medium, with no significant differences observed between samples, suggesting that none of these materials trigger cell membrane damage at this time point. However, after 7 days of culture, the LDH activity was enhanced in both analysed samples, with a significant increase in the control sample, as compared with the PLA/HAp/BMP4 materials, which sustain the enhanced cytotoxicity of the non-coated surface. More, correlated with the cell viability assays results that sustain the excellent biocompatibility of the PLA/HAp/BMP4 coatings, the increase of the LDH activity in the media samples collected from cells-PLA/HAp/BMP4 bioconstructs could be attributed to the prolonged cell culture period, as well as from natural cell death process as a result of the rapid cell growth on the material surface that reached monolayer confluence within 1-week.

**Figure 9:**
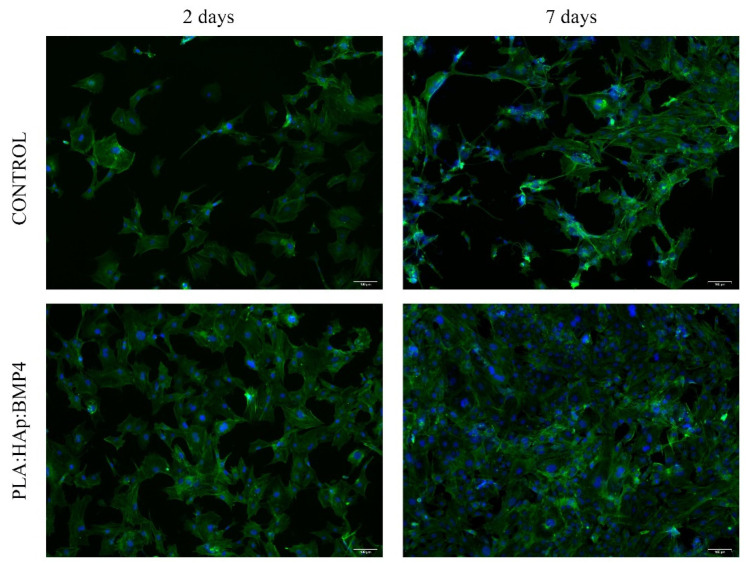
Confocal microscopy images revealing the MC3T3-E1 cytoskeleton’s actin filaments (green fluorescence) and MC3T3-E1 cells nuclei (blue fluorescence), after 2 and 7 days of culture of contact with the control surface and PLA/HAp/BMP4 coatings (scale bar 100 μm).

To investigate the effects of the investigated samples on MC3T3-E1 cellular morphology, fluorescence microscopy was employed after fluorescent staining with FITC-phalloidin for cytoskeleton’s actin filaments and DAPI for cells nuclei (Figure 9). After 2 days of culture, the MCT3-E1 cells cultured in contact with the control samples were majority round-shaped and exhibited short actin filaments, while the preosteoblasts cultured in contact with the PLA/HAp/BMP4 coatings were spindle-shaped with long, well-defined actin filaments, features that suggest a tight adhesion of cells to the nanostructured surface. After 7 days, MC3T3-E1 cells also adopted their fibroblast-like morphology on the control surface, but round cells, with little actin, condensed around the nuclei were still noticed. The cells were erratic, scattered on the control surface and agglomerated in cell clusters, leaving a large area of the material unpopulated, probably due to poor cell motility and proliferative potential. In contrast, 3T3-E1 cells cultured onto PLA/HAp/BMP4 nanostructured surfaces covered the entire material surface and formed a compact cellular network, distributed evenly onto the material surface.

### 3.4. In Vitro Osteoinductive Potential of PLA/HAp/BMP4 Coatings

**Figure 10:**
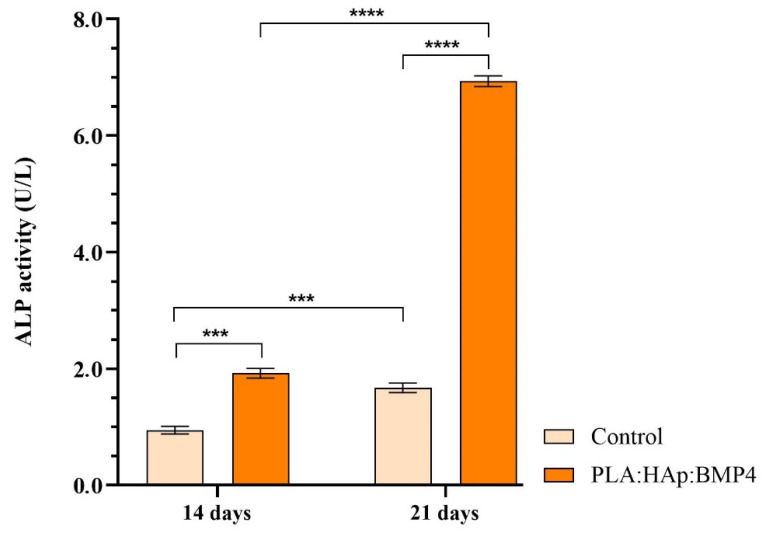
ALP activity in culture media harvested from 3T3-E1 cells cultured in contact with control samples and PLA/HAp/BMP4 coatings and osteogenic inductors for 3 weeks (*** *p* < 0.001; **** *p* < 0.0001).

MC3T3-E1 pre-osteoblasts, cultured on control samples and PLA/HAp/BMP4 coatings, were exposed to osteogenic inductors for 21 days. Various assays were employed to monitor several features specific to the osteogenic differentiation process. In this view, after 14 and 21 days, the activity of ALP was determined as being one of the earliest markers of mature osteoblasts (Figure 10). As revealed by the ascending ALP activity during 21 days of exposure to osteogenic inductors, both analyzed samples increased the activity of ALP. However, between cell culture time points, the ALP activity was 3.6-fold increased at 21 days vs. 14 days in the media samples collected from PLA/HAp/BMP4 coatings, as compared with 1.7-fold increase determined in the reference samples.

**Figure 11:**
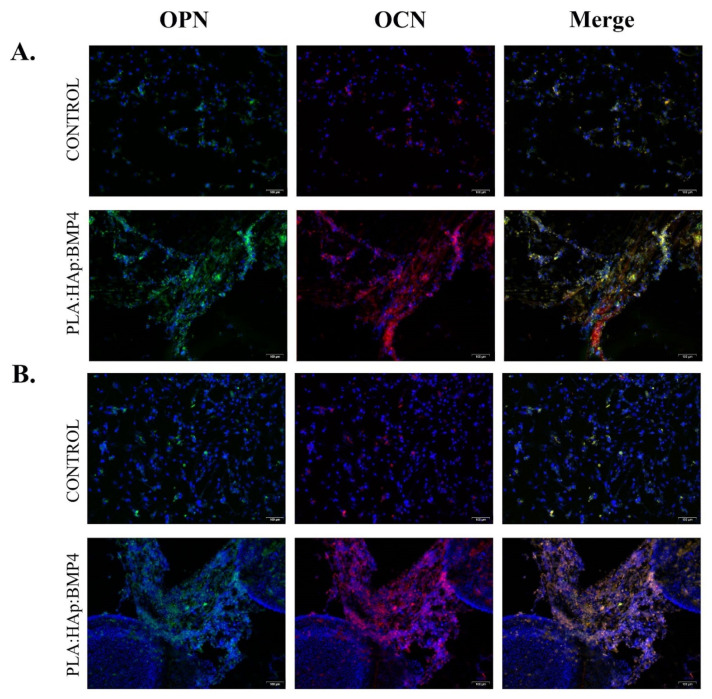
Protein expression of osteogenic specific markers OPN (green) and OCN (red), as revealed by fluorescence microscopy, after 14 (**A**) and 21 (**B**) days of osteogenic differentiation induction of MC3T3-E1 cells on control surfaces and PLA/HAp/BMP4 coatings (scale bar 100 μm). The cell nuclei are stained with DAPI (blue).

Moreover, as the ALP activity is tightly correlated with the expression of osteopontin (OPN) and osteocalcin (OCN), key player molecules in the biological and mechanical functions of bone and secreted by active osteoblasts, the protein expression of these late-osteogenic markers was investigated by fluorescence microscopy (Figure 11). After 14 days of osteogenic induction of MC3T3-E1, cultured on reference surfaces and PLA/HAp/BMP4 coatings, the positive expression of OPN and OCN was observed in both samples. However, the ratio of MC3T3-E1 cells, cultured onto control surface expressing OPN and OCN, was significantly lower than the ratio of MC3T3-E1 cultured onto PLA/Hap/BMP4 coatings, where almost all cells had various amount of OPN and OCN. On the nanostructured coatings MC3T3-E1, expressing high amounts of OPN and OCN, cells organized in a 3D cellular network, the expression of the osteogenic markers being significantly increased at the level of intercellular junctions. After 21 days of osteogenic induction, no changes in the OPN and OCN protein expression were observed for MC3T3-E1 cells cultured in contact with the reference slides. Concerning the PLA/HAp/BMP4 coatings, an enhancement of the OPN and OCN protein expression was noticed, compared with the 14-days expression levels. A limited amount of MC3T3-E1 separate from the compact cellular network and condensate to generate 3D cellular spheroids that lack the expression of OPN or OCN, most probably because cells joining the condensation phase lose their active state and turn into osteocytes.

**Figure 12:**
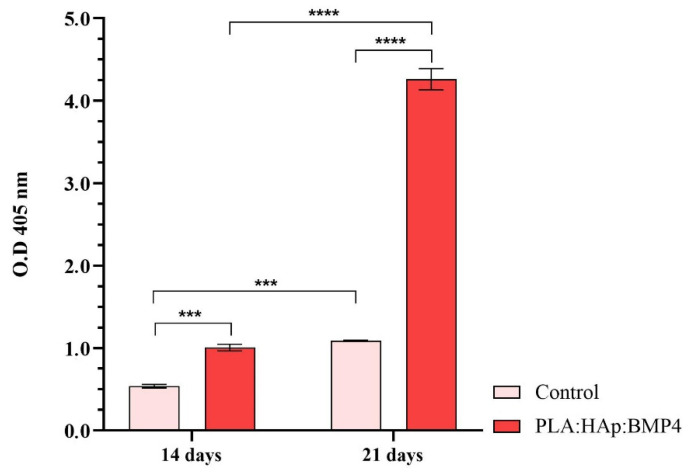
Quantification of the alizarin red S staining after 14 and 21 days of MC3T3-E1 osteogenic differentiation in contact with control samples and PLA/HAp/BMP4 coatings (*** *p* < 0.001; **** *p* < 0.0001).

In the end, to investigate the amount of calcium deposits generated after exposure of MC3T3-E1 to control surfaces and PLA/HAp/BMP4 coatings and pro-osteogenic stimuli, alizarin red S staining was performed (Figure 12). The obtained results showed that moderate mineralization of the extracellular matrix process is present in both experimental conditions after 14 days of culture, a greater amount of calcium deposits being present in MC3T3-E1 cells cultured onto PLA/HAp/BMP4 coatings. While the amount of calcium deposits increases significantly after 21 days of culture, in both MC3T3-E1-material samples, in comparison with 14 days of culture, the obtained results highlight a poor performance of the reference sample to sustain the mineralization of the extracellular matrix, as the level of alizarin red S staining are 4-fold lower to the level identified for MC3T3-E1- PLA/HAp/BMP4 coatings bioconstructs at 21 days.

## 4. Conclusions

In this study, the MAPLE technique was employed to obtain coatings based on materials that already showed great potential in bone tissue engineering (PLA, HAp), which were blended with BMP4, a growth factor that induces the osteogenic differentiation of osteoblasts and osteoprogenitors and promotes bone formation to improve the bioactivity of the metallic implants. Independent of the targeted medical application, a crucial aspect is to obtain a biocompatible material, this aspect was investigated using the preosteoblast MC3T3-E1 cell line by investigating the cell behavior and health, in contact with the PLA/HAp/BMP4 coatings. The obtained results showed that, when using the PLA/HAp/BMP4 coatings for substrates, an excellent biocompatible material is obtained, which sustains cell adhesion, viability, and proliferation and lacks cytotoxicity. This excellent biocompatibility was attributed to the fact that the individual components (PLA, HAp) used for synthesizing the hybrid nanostructured coatings are intensively used in biomedical applications for good biocompatibility, besides other mechanical advantages. The osteogenic potential of the PLA/HAp/BMP4 coatings was investigated to assess the capacity of the nanostructured surfaces, in order to improve the in vitro osteogenic response of MC3T3-E1 preosteoblasts, after exposure to osteogenic inductors, by evaluation of ALP activity, an early marker of osteoblast phenotype, as well as late markers, such as the bone matrix proteins expression (OPN, OCN), which are by activated osteoblasts and extracellular matrix mineralization and condensation, as final steps of the osteogenic differentiation pathway. Investigation of all these crucial features for the osteogenic differentiation pathway showed that, in the absence of the PLA/HAp/BMP4 coatings, preosteoblasts present a poor yield, regarding osteogenic differentiation, despite the pro-osteogenic inductors provided. In contrast, combining materials with well-known osteoinductive proprieties proved to be an excellent strategy for obtaining a novel biocompatible osteoinductive coating that can improve the currently available implants for bone tissue engineering applications. The results of this study support the use of PLA/HAp/BMP4 coatings in future studies, to assess the ability of these nanostructured materials in supporting the repair process of bone damage
